# A Comparative Evaluation of the Effects of Herbal Denture Adhesives on Patient Comfort and Satisfaction: Study Protocol of a Randomized Controlled Trial

**DOI:** 10.7759/cureus.66276

**Published:** 2024-08-06

**Authors:** Pooja Chitlange, Seema R Kambala, Surendra Agrawal, Anjali Bhoyar, Vedant Pathak, Pragati Agarwal

**Affiliations:** 1 Department of Prosthodontics and Crown and Bridge, Sharad Pawar Dental College and Hospital, Datta Meghe Institute of Higher Education and Research, Wardha, IND; 2 Department of Pharmacy, Datta Meghe College of Pharmacy, Datta Meghe Institute of Higher Education and Research, Wardha, IND

**Keywords:** denture adhesive, mesoporous nanoparticles, complete denture, essential oil, herbal denture adhesive

## Abstract

Introduction

The lack or complete loss of all-natural teeth is known as edentulism, and it happens frequently. One of the most prevalent issues among edentulous patients is severe resorption of the residual alveolar ridge, which can still occur even with cautious prosthetic treatment. Therefore, one of the biggest issues faced in dentistry today is retention and stability in the rehabilitation of completely edentulous patients using complete denture prostheses. This problem was resolved by using denture adhesives. Studies show that when denture adhesive is applied correctly, it improves stability and effectiveness for individuals wearing complete dentures by increasing the surface tension between the alveolar mucosa and the denture fitting surface. The denture adhesive must possess safety, cost-effectiveness, and sufficient antibacterial and antifungal properties. The application should be easy, without causing any mess, and have a pleasant smell. It should not alter or obstruct the denture's intaglio surface. Nowadays, it is acknowledged that denture adhesives are treatments that can be used in association with dentures.

Methods

Based on the type of denture adhesive, the patients will be randomly divided into two groups, i.e., 20 patients in each group: Group A (herbal denture adhesive without essential oil) and Group B (herbal denture adhesive with essential oil). The patients will be requested to fill out the questionnaire for masticatory efficiency, retention, stability, etc., at the time of denture insertion without using denture adhesive and seven days after the use of denture adhesive.

Expected results

The overall satisfaction of complete denture patients will be significantly increased when an herbal denture adhesive infused with essential oil is used. This will result in increased stability, retention of the mandibular denture, improved masticatory efficiency, and patient satisfaction.

Conclusions

Patients who are elderly may experience physical and psychological challenges when adjusting to new or relined dentures. Denture adhesive, however, can aid in the patient's satisfaction with the prosthesis' altered occlusion and fit. It also makes the patient feel more confident and secure. A dental professional must be consulted before using denture adhesive.

## Introduction

Edentulism, defined as the absence or total loss of natural teeth, is a common occurrence. Severe alveolar ridge atrophy is a common problem in edentulous patients, and it can occur even in the case of meticulous prosthesis management. As a result, one of dentistry's biggest concerns is the rehabilitation of completely edentulous individuals. A complete denture prosthesis is the most commonly used method for the rehabilitation of completely edentulous patients. Retention and stability are the biggest challenges faced by the dentist in the case of resorbed and compromised ridges; dentists address this issue by using denture adhesive to enhance denture adherence and retention [1]. Studies show that when denture adhesive is applied correctly, it improves stability and effectiveness for individuals wearing complete dentures by raising the surface tension between the alveolar mucosa and the denture fitting surface. Denture adhesives need to be strong, reasonably priced, and possess adequate antibacterial and fungal properties. It should also flavor and smell excellent, be easy to use and obtain, and should not change or damage the intaglio surface of the denture [2]. It is increasingly recognized that denture adhesives are treatments that can be utilized in addition to dentures. A denture adhesive can help an older patient acclimatize to the changing shape, fit, and occlusion of their prosthesis, as it can be difficult for them to adjust both physically and emotionally to new or relined dentures. The patient feels more secure and content when using denture adhesive. However, it is imperative to consult with a dental professional before using denture adhesive [3].

Denture adhesives are categorized based on their forms, such as powder, tapes, gels, and paste. Denture adhesive enhances retention, lessens denture soreness, avoids strangling the mucosal blood supply, and lessens the need for adjustments even when the denture fits [4]. Additionally, there have been reports of improvements in biting force and chewing efficiency, which lead to a wider dispersion of occlusal forces over the tissues supporting the denture and a decrease in local pressure. The lubricating and cushioning properties of adhesives lessen friction and discomfort in the mucosa. Denture adhesives can be very beneficial for patients with muscle control deficiencies caused by hormonal or neurotransmitter abnormalities [5]. This study aims to evaluate and compare two different types of commercially available herbal denture adhesives with regard to patient satisfaction with denture retention.

## Materials and methods

Method

This experimental study will be conducted in the Department of Prosthodontics, Sharad Pawar Dental College and Hospital, Sawangi (M), Wardha, India. The data will be collected in the postgraduate clinic of the Department of Prosthodontics. On the basis of the inclusion and exclusion criteria (Table 1), the patients will be divided randomly into two groups: Group A (n=20) (herbal denture adhesive without essential oil) and Group B (n=20) (herbal denture adhesive with essential oil). A total of 16 closed-ended questions will be asked of patients: eight during denture insertion without using denture adhesives and eight questions seven days after using denture adhesives.

**Table 1:** Inclusion and exclusion criteria

Inclusion Criteria	Exclusion Criteria
Patients who are first-time denture wearers	Patients with tooth-supported overdentures
Patients who are old complete denture wearers	Patients with implant-supported overdentures
Patients who are willing to participate in the study	Patients with a single complete denture

The denture adhesive will be fabricated by utilizing carboxymethylcellulose with flavor, mineral oil, petroleum, silica particles, and mesoporous nanoparticles of the selected essential oil (cinnamon oil) with antimicrobial action incorporated into a gel for prolonged release, giving better effectiveness. In order to gather information about denture fitting, removal, chewing, retention, and phonetics, a standard questionnaire form will be given to each patient. Following the patient's answer to Questionnaire 1 (Table 2), a clinical examination and application of denture adhesive demonstration will take place, adhering to the guidelines provided by the manufacturer. The patients will be provided with an explanation of the cleaning routine and the administration of denture adhesive for the next seven days. The patients will be contacted back a week later to complete an additional questionnaire (Table 3) in which they will be asked to rate the denture's strength, biocompatibility, convenience, and masticatory abilities using the glue that was provided.

**Table 2:** Questionnaire 1 (at the time of denture insertion) Reference: [1,6,7]

Sr. No.	Questions	Options	No. of Patients
1.	To what extent do you find the retention of your upper denture satisfactory?	Dissatisfied	
		Neutral	
		Satisfied	
2.	To what extent do you find the retention of your lower denture satisfactory?	Dissatisfied	
		Neutral	
		Satisfied	
3.	To what extent do you find the chewing ability of your denture satisfactory?	Dissatisfied	
		Neutral	
		Satisfied	
4.	To what extent are you happy with the comfort of your new denture?	Dissatisfied	
		Neutral	
		Satisfied	
5.	To what extent do you find the speech and sound produced by the denture satisfactory?	Dissatisfied	
		Neutral	
		Satisfied	
6.	How satisfied are you with the way your dentures fit and removal?	Dissatisfied	
		Neutral	
		Satisfied	
7.	Does your denture harm the soft tissues in any way?	Yes	
		No	
8.	Do you have any issues with swallowing because of the denture?	Yes	
		No	

**Table 3:** Questionnaire 2 (after seven days of denture insertion) Reference: [1,6,7]

Sr. No.	Questions	Options	No. of Patients
1.	To what extent are you satisfied with the adhesive's ability to keep your upper denture in place?	Dissatisfied	
		Neutral	
		Satisfied	
2.	To what extent are you satisfied with the adhesive's ability to keep your lower denture in place?	Dissatisfied	
		Neutral	
		Satisfied	
3.	How was your chewing experience after using this denture adhesive?	Worse	
		No difference	
		Better	
4.	For what duration did the denture adhesive work on your dentures?	≤4 hours	
		4-8 hours	
		8-12 hours	
5.	How does this denture adhesive taste?	Bad	
		Neither good nor bad	
		Good	
6.	How did you find it to remove the adhesive from your dentures?	Difficult	
		Neutral	
		Easy	
7.	How did you find it difficult to clean your denture after using the denture adhesive?	Difficult	
		Neutral	
		Easy	
8.	How difficult was it for you to wipe your oral cavity after using the denture adhesive?	Difficult	
		Neutral	
		Easy	

Sample Size

The sample size is determined using the following formula: n = {(Z 1-α/2 + Z 1-β)2 p1(1-p1) + p2 (1-p2)}/(p1-p2)^2^, where the proportion of outcome from group 1 (p1) = 0.37, the proportion of outcome from group 2 (p2) = 0.22, the level of significance (α) = 0.05, power (1-β) = 0.80, Z alpha value = 1.96, Z beta value = 0.84, and the total sample size = 40. The sample size estimation is done at a 95% confidence interval. Each group consists of 20 samples.

Statistical analysis

The statistical analysis will be done using IBM SPSS Statistics for Windows, Version 23 (Released 2015; IBM Corp., Armonk, New York). Descriptive statistics will be used for the mean distribution of both groups, and an independent sample t-test will be used for intergroup analysis between both groups.

Age will be given using mean and standard deviation; however, frequency percentage will be used for qualitative characteristics. The independent t-test will be used to compare age between the two groups, and the Pearson chi-square test or Fisher's exact test will be used to compare all qualitative variables at a significant level of p-value ≤0.05 for all variables. The total of each questionnaire's scores will be used to determine the overall satisfaction with the results. Next, choose the percentage of satisfaction for every patient in both groups. The Wilcoxon signed-rank test will be used to compare patient satisfaction before and after the interview.

Ethical considerations

This study has been approved by the Institutional Ethics Committee of Datta Meghe Institute of Higher Education and Research (DMIHER(DU)/IEC/2024/56) dated May 11, 2024. Participant confidentiality will be maintained, and all procedures will adhere to ethical guidelines for research.

Trial registration

The study is registered with the Clinical Trial Registry-India under the CTRI Registration number CTRI/2024/06/068933, registered on June 14, 2024.

## Results

The current study's findings will indicate that the application of herbal denture adhesive combined with essential oil will considerably improve the overall comfort of using the complete denture. It will reduce the bacterial growth and chances of mouth sores due to dentures. The expected results of the study indicate that the utilization of herbal denture adhesive combined with essential oil will increase the stability and retention of the dentures and the effectiveness of their retention over time.

## Discussion

Denture adhesives are categorized based on how they are manufactured, such as powder, tapes, gels, and paste. Denture adhesive enhances retention, lessens denture soreness, avoids strangling the mucosal blood supply, and lessens the need for adjustments even when the denture fits.

A study by Mekkawy et al. involved 30 edentulous patients who were divided into two groups and given various types of denture adhesives (SECURE and COREGA) for seven days. The patients' satisfaction with denture retention, chewing ability, stability, and efficiency were assessed. The study found that the prosthetic denture adhesives significantly improved denture retention in the mandible and the duration of the adhesion in the mouth. However, there was no significant increase in the ability to remove the adhesive or maxillary denture retention. The study suggests that effective use of denture adhesives requires patient education and proper dental guidance [1].

A study by Yadav et al. involved 30 edentulous patients with resorbed ridges. They used isabgol, fixon powder, and Fittydent powder denture adhesives. Incisal bite force and retention force were measured using FlexiForce sensors (Tekscan, Massachusetts) and digital force gauges. Fittydent powder and isabgol significantly increased retention and biting force compared to fixon [4].

A study by Koronis et al. involved 30 edentulous patients who had their denture-supporting tissues and old dentures renewed. They were prescribed three different brands of denture adhesives: Fittydent, Seabond, and Protefix. The adhesives were used for 48 hours on the lower denture, with participants spending 24 hours without adhesives. The results showed that denture adhesives generally enhance patient satisfaction and chewing ability, especially in those with poor Kapur index and poor denture retention. Fittydent was found to be the most effective adhesive [8].

A study by Ereifej et al. surveyed 64 edentulous patients for the first time, comparing their satisfaction with denture adhesives. The patients were divided into three groups, each receiving a complete denture prosthesis. After a month, they rated their satisfaction on a 100 mm Visual Analog Scale (VAS) scale and completed an oral health impact profile questionnaire. The study found that all groups had significantly higher VAS values and lower OHIP-EDENT scores after applying the adhesives. However, the ease of cleaning was significantly different between groups. The study concluded that denture adhesives improved patient satisfaction and standard of living [9].

Study limitation

The present study is conducted to create a novel herbal denture glue that contains essential oil and can reduce bacterial count while also being more advantageous to patients in retention, stability, enhancing mastication, and esthetics. However, the study's sample size and short duration limit the generalizability of the findings. Further research with larger sample sizes and longer follow-up periods is recommended to confirm these results and assess long-term effects.

## Conclusions

Patients who are elderly may experience physical and psychological challenges when adjusting to new or relined dentures. Denture adhesive, however, can aid in the patient's acclimatization to the prosthesis. It also makes the patient feel more confident and secure. A dental professional must be consulted before using denture adhesives.
